# Cefepime versus carbapenems for treatment of AmpC beta-lactamase-producing Enterobacterales bloodstream infections

**DOI:** 10.1007/s10096-023-04715-5

**Published:** 2023-11-23

**Authors:** Julia Herrmann, Anne-Valérie Burgener-Gasser, Daniel Goldenberger, Jan Roth, Maja Weisser, Pranita D. Tamma, Sarah Tschudin-Sutter

**Affiliations:** 1 Division of Infectious Diseases and Hospital Epidemiology, University Hospital Basel and University of Basel, Petersgraben 4, CH-4031 Basel, Switzerland; 2 Division of Clinical Bacteriology and Mycology, University Hospital Basel and University of Basel, Basel, Switzerland; 3 Department of Pediatrics, Johns Hopkins University School of Medicine, Baltimore, MD USA

**Keywords:** AmpC beta-lactamase, Enterobacterales, Cefepime, Carbapenem-sparing regimen, Bloodstream infection

## Abstract

**Purpose:**

Cefepime is recommended for treating infections caused by AmpC beta-lactamase-producing Enterobacterales (AmpC-PE), though supporting evidence is limited. Therefore, this study compared outcomes associated with cefepime versus carbapenem therapy for bloodstream infections (BSIs) caused by AmpC-PE after phenotypic exclusion of ESBL-co-producing isolates.

**Methods:**

This retrospective cohort study compared definite cefepime versus carbapenem treatment for AmpC-PE BSI in hospitalized patients of the University Hospital Basel, Switzerland, between 01/2015 and 07/2020. Primary outcomes included in-hospital death, renal impairment and neurologic adverse events; secondary outcomes included length of hospital stay and recurrent infection.

**Results:**

Two hundred and seventy episodes of AmpC-PE BSI were included, 162, 77 and 31 were treated with a carbapenem, cefepime and other antibiotics, respectively. Patients treated with carbapenems were more likely to be transferred to the ICU on admission and more frequently had central venous catheter as a source of infection. In uni- and multivariable analyses, primary and secondary outcomes did not differ between the two treatment groups, except for more frequent occurrence of neurological adverse events among patients treated with carbapenems and shorter length of hospital stay among survivors treated with cefepime.

**Conclusion:**

After excluding isolates with phenotypic ESBL-co-production, cefepime was not associated with adverse outcomes compared to carbapenems when used to treat BSIs caused by AmpC-PE. Our study provides evidence to support the use of cefepime as a safe treatment strategy for AmpC-PE BSI, particularly in clinically stable patients without initial renal impairment or increased susceptibility to neurological adverse events.

**Supplementary Information:**

The online version contains supplementary material available at 10.1007/s10096-023-04715-5.

## Introduction

Increasing resistance of Enterobacterales to beta-lactam antibiotics, especially to carbapenems, is a global concern [1, 2]. Thus, carbapenem-sparing treatment strategies should be prioritized when there are alternative agents with evidence to support their safety and efficacy.

The Infectious Diseases Society of America (IDSA) recommends the use of cefepime for the treatment of infections caused by Enterobacterales at moderate to high risk of significant AmpC production, such as *Enterobacter cloacae*, *Klebsiella aerogenes,* and *Citrobacter freundii –* particularly for those isolates with minimal inhibitory concentrations (MICs) of 2 µg/ml or less for cefepime to reduce the likelihood of extended spectrum beta-lactamase (ESBL) co-production [3]. However, the guidance document acknowledges the lack of evidence supporting their recommendation, which is based on observational data with limited sample sizes [4–6]. The guideline for treatment of multidrug-resistant gram-negative bacilli published by the European Society of Clinical Microbiology and Infectious Diseases (ESCMID) acknowledges a very low certainty of evidence regarding the non-inferiority of cefepime as compared to carbapenems for treatment of AmpC-producers [7].

Cefepime, a fourth generation cephalosporin with enhanced stability against AmpC-producing Enterobacterales, has favorable pharmacokinetic and pharmacodynamic properties when its dosing is optimized [8, 9]. Despite its activity against AmpC, cefepime is not stable to extended-beta-lactamases (ESBL) which can be co-expressed by Enterobacterales [10, 11], leading to clinical failures [10, 11]. Cefepime has also been associated with neurotoxicity, particularly in patients with impaired renal function; carbapenems are often selected over cefepime to avoid these neurologic effects [12, 13]. As of yet, there are no randomized clinical trials evaluating cefepime for the treatment of infections caused by AmpC-producing Enterobacterales (AmpC-PE); therefore, we aim to contribute to the available literature by comparing clinical outcomes associated with cefepime versus carbapenem therapy for bloodstream infections (BSI) caused by AmpC-PE.

## Materials and methods

### Study design and setting

This was a retrospective observational cohort study conducted at the University Hospital Basel (USB), a Swiss tertiary care center with over 35,000 hospital admissions per year, to compare definitive cefepime versus carbapenem therapy for BSI caused by AmpC-PE. Enterobacterales were classified as AmpC-producers based on species identification and no further confirmatory testing for presence of AmpC was performed. Study participants were identified by systematically extracting all consecutive patients with blood cultures positive for AmpC-PE from the electronic database of the Microbiology Laboratory between January 1^st^, 2015 and July 31^st^, 2020. Hospitalized patients ≥ 18 years of age with AmpC-PE BSI and receiving definite therapy with either cefepime or a carbapenem for AmpC-PE BSI were included Patients were excluded if they were transferred to another hospital within 48 h of receipt of the positive blood culture result, if antibiotics were withheld per recommendation of the palliative care team, or if they died prior to receipt of the study drug. All positive blood cultures are reviewed by the infectious disease consultation service on a daily basis (including weekends) at our institution, and local treatment guidelines discourage cefepime use if renal function is impaired (creatinine clearance < 30 mL/min). Our guidelines point to cefepime’s potential to cause adverse events affecting the central nervous system, including lowering the seizure threshold [14]. The standard dosing of cefepime was 2 g twice daily, dosing was reduced if renal function was impaired (i.e. 1 g three times daily for a creatinine clearance between 40-70 ml/min, 1 g twice daily for a creatinine clearance between 10-40 ml/min, and 1 g once daily for a creatinine clearance < 10 ml/min). The standard dosing of meropenem was 1 g three times daily, dosing was reduced if renal function was impaired (i.e. 1 g twice daily for a creatinine clearance between 26-50 ml/min, 500 mg twice daily for a creatinine clearance between 10-25 ml/min and 500 mg once daily for a creatinine clearance between < 10 ml/min). The standard dosing of ertapenem was 1 g once daily, dosing was reduced to 500 mg once daily if creatinine clearance was below 30 ml/min. The standard dosing of Imipenem/Cilastatin was 500 mg four times daily, dosing was reduced if renal function was impaired (i.e. 500 mg three times daily for a creatinine clearance between 30-50 ml/min, 500 mg twice daily for a creatinine clearance between 10-30 ml/min and 250 mg twice daily for a creatinine clearance between < 10 ml/min).

This study is registered at ClinicalTrials.gov with no deviation from the overall protocol (NCT04577989) and approval by the local ethics committee was obtained (EKNZ-2020–02251). We adhered to the Strengthening the Reporting of Observational Studies in Epidemiology (STROBE) guidelines [15].

### Data collection and outcomes

Pertinent clinical data was collected from electronical medical records and encoded in a secure web-based REDCap database [16]. The primary outcomes included in-hospital all-cause mortality, new renal impairment, and new neurologic adverse events. We considered all three events as clinically important and therefore choose to define them all as primary outcomes. Secondary outcomes included length of hospital stay – overall and among survivors only—as well as recurrent infections with AmpC-PE during the following six months of the initial episode.

The following data were collected: demographics: age, gender, hospital admission and discharge, length of stay, ICU-stay, hospitalization prior to current hospital stay, discharge destination, outcome and cause of death; comorbidities based on the Charlson Comorbidity Index and Elixhauser Comorbidity Score; renal function during hospitalization; antibiotic therapy within the previous 3 months (as determined by reviewing patient’s in-and outpatient records, if available); immunosuppressing medications (as defined below) or immunocompromising condition within the prior 12 months; evidence and type of recurrent infection with AmpC-producing Enterobacterales in the following six months; ICU transfer within 24 h of AmpC-PE BSI onset and need of vasopressors or mechanical ventilation; maximum body temperature within 12 h of blood culture collection; source of infection and presence of source control (source control was defined as drainage/debridement of abscesses and/or wounds, removal of hardware combined with clinical signs of improvement, such as cessation of fever, declining inflammatory markers and no signs of residual infection in imaging if available); results of antimicrobial susceptibility test and emergence of pathogen resistance to study drug during antibiotic therapy, *Clostridioides difficile* infections in the following six months, dates of initiation, discontinuation, and changes in antibiotic therapy, adverse events attributed to antibiotic therapy reported during hospital stay.

The collection date of the positive blood culture was the reference time point for subsequent time specifications, including infection onset. ESBL-production was suspected based on the detection of resistance to cefpodoxime, ceftriaxone, ceftazidime, or aztreonam. Phenotypic confirmation of the ESBL-PE was performed by Etest® strips (bioMérieuex, Marcy-l’Etoile, France) using cefotaxime, ceftazidime, or cefepime, each tested with and without clavulanic acid or with ROSCO disks (Rosco, Taastrup, Denmark).

### Definitions

Recurrent infections included any infection with AmpC-PE within a follow-up period of six months after diagnosis of an initial AmpC-PE episode. Immunosuppressive treatment or condition within the past 12 months was defined as chemotherapy in the last 6 months, untreated or insufficiently treated (CD4 cell count < 500/μL) HIV infection as well as primary immunodeficiency or immunomodulatory therapy (glucocorticoids, calcineurin inhibitors, mTOR inhibitors, cytostatics, monoclonal antibodies, mycophenolate) in the last 30 days or prednisone > 10 mg (or equivalent) for > 14 days within the last 30 days [4].

Empiric antibiotic treatment was defined as the antibiotic regimen administered prior to the diagnosis of AmpC-PE infection while identification and susceptibility testing results were pending [17]. The susceptibility of pathogens was classified according to the EUCAST criteria valid during the study period. The revised EUCAST definitions of susceptibility testing categories was introduced after the study period in autumn 2020, therefore, we considered the intermediate category as resistant within the clinical context [18].

An empiric antibiotic treatment to which the identified pathogen was found non-susceptible was defined as inadequate. Definitive antibiotic treatment applied to the first administered antibiotic regimen after confirmation of AmpC-PE and receipt of susceptibility test results from blood cultures. All AmpC-PE were confirmed susceptible to the definitive treatment regimens.

Renal function was expressed as glomerular filtration rate (GFR) and categorized using the KDIGO classification [19]. The degree of acute kidney injury (AKI) during antibiotic therapy was defined according to the KDIGO Acute Kidney Injury Work Group as meeting criteria for stage 1, stage 2 and stage 3 [20]. New onset renal impairment was considered a KDIGO-defined AKI in patients with no baseline renal dysfunction or an increase of the KDIGO class by at least one category in those already meeting KDIGO criteria at baseline.

Neurological adverse events included all reported events of an epileptic seizure, an encephalopathic state or delirium during the treatment period for AmpC-PE BSI as diagnosed by a physician and recorded as a diagnosis in the medical record.

Further details on microbiological characteristics of AmpC-PE BSI stratified by definite antimicrobial therapy and AmpC-PE are included in the supplementary material.

### Statistical analysis

Categorical and numerical variables were compared using the Fisher’s exact and Wilcoxon rank-sum test, respectively. Crude and adjusted associations between definite antimicrobial therapy and binary outcomes (i.e. in-hospital death, recurrent infection, new renal impairment, and neurological adverse events) were estimated using logistic regression models. For the outcome length of stay, we fitted generalized linear models with a log link and gamma distribution. Based on baseline differences between exposures (the groups were already quite balanced at baseline) and data sparsity/multicollinearity, exposure-outcome associations were adjusted for ICU admission within 24 h and the following source of infection: skin/soft tissue, abdomen, and central venous catheters. All models were based on complete cases. As 13 patients contributed twice and two patients contributed to three episodes during the study period resulting in 224 (patient) clusters overall, a sensitivity analysis was performed for the different exposure-outcome associations using cluster robust standard errors. All analyses were performed on a multicore system with Stata/MP version 16 (Stata Corp., College Station, Texas, United States). Reported P-values are two-sided.

## Results

### Cohort and microbiological data

**Fig. 1:**
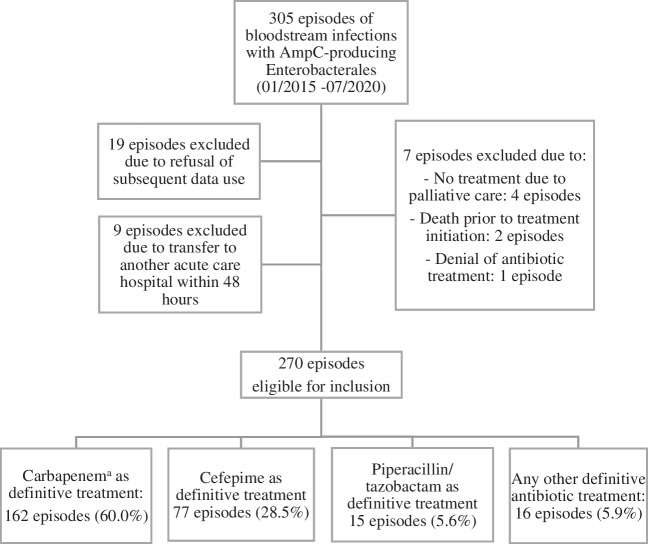
Flow chart of case selection; ^a^meropenem, imipenem, or ertapenem combined

Within the study period, 305 episodes of BSI caused by AmpC-PE were identified among 288 patients, of which 270 episodes met eligibility criteria. Reasons for exclusion are summarized in Fig. 1. Carbapenems were administered as definitive antimicrobial treatment in 162 episodes, cefepime in 77 episodes, piperacillin/tazobactam in 15 episodes and other antibiotic agents in 16 episodes (Fig. 1). Therefore, 239 episodes were included in the comparative analysis between carbapenems and cefepime. Twelve BSI episodes were polymicrobial, 11 with two different AmpC-PE and one with three different AmpC-PE, resulting in 283 AmpC-PE isolates identified.

Among the 283 AmpC-PE isolates, 47.3% were identified as *Enterobacter cloacae* group, 27.4% as *Serratia marcescens*, 14.4% as *Klebsiella aerogenes*, 10.4% as *Citrobacter freundii* group and 7.0% as *Morganella morganii*. ESBL-production was confirmed in six out of the 283 total AmpC-PE isolates (2.1%) and none produced a carbapenemase (Supplementary table 1). Forty-eight AmpC-PE (16.9%) were intermediate or resistant to piperacillin/tazobactam, 35 (12.5%) were intermediate or resistant to any carbapenem [17 (6.1%) against imipenem, 15 (5.3%) against ertapenem and three (1.1%) against meropenem only] and 20 (7.0%) were intermediate or resistant to cefepime (Supplementary table 1).

### Baseline characteristics

Comparisons of baseline characteristics and risk factors between patients treated with carbapenems and cefepime are presented in Table 1. Patients treated with carbapenems were more likely to be transferred to the ICU within 24 h (35.0% vs 11.8%; *p* < 0.001) and more frequently required vasopressors (23.8% vs 7.9%; *p* = 0.004) or mechanical ventilation (21.9% vs 7.9%; *p* = 0.009). Central venous catheter-associated infections were more frequent in the carbapenem group (18.1% vs 3.9%, *p* = 0.002). Both groups were well balanced in terms of comorbidity scores (median 3.0 vs 2.0, *p* = 0.734) and receipt of adequate empirical antibiotic therapy (77.2% vs 79.2%, *p* = 0.868).

**Table 1:** Baseline characteristics stratified by definite antimicrobial therapy

	Definite carbapenem treatment (*n* = 162)		Definite cefepime treatment (*n* = 77)		*p*-value	Missing data (*n* = 239)
	n/ median	%/ IQR	n/ median	%/IQR		n / %
Demographics						
Age	70.5	59.0–79.0	70.0	60.5–78.0	0.923	0 / 0.0
Female gender	59	36.4	25	32.5	0.566	0 / 0.0
Admission from					0.297	0 / 0.0
Home	103	63.6	58	75.3		
Acute care hospital (Switzerland)	45	27.8	12	15.6		
Long-term care facility	6	3.7	4	5.2		
Nursing home	3	1.9	2	2.6		
Residency abroad	4	2.5	1	1.3		
Acute care hospital abroad	1	0.6	0	0.0		
Ward group					0.276	0 / 0.0
Surgery	61	37.7	21	27.3		
Medicine	74	45.7	42	54.5		
Other	27	16.7	14	18.2		
Comorbidities						
Charlson Comorbidity Index Score	3.0	1.0–4.0	2.0	2.0–4.5	0.734	0 / 0.0
Elixhauser Comorbidity Score	10.0	4.0–15.0	9.0	4.5–13.0	0.782	0 / 0.0
Solid organ transplantation	5	3.1	3	3.9	0.715	0 /0.0
Stem cell transplantation	5	3.1	3	3.9	0.716	1 / 0.4
Exposures						
Previous antibiotic treatment ^a^	102	69.4	42	60.0	0.219	22 / 9.2
Previous PPI therapy ^a^	101	62.7	47	61.8	0.887	2 / 0.8
History of immunosuppression ^b^	41	25.3	25	32.5	0.279	0 / 0.0
Microbiological history						
Colonization or infection with any resistant pathogen ^c^	12	7.4	4	5.2	0.593	30 / 12.5
ESBL-producing bacteria	9	5.6	2	2.6		
Carbapenemase-producing bacteria	0	0.0	0	0.0		
Vancomycin-resistant Enterococcus	0	0.0	0	0.0		
Methicillin-resistant *Staphylococcus aureus*	1	0.6	0	0.0		
Others	3	1.9	2	2.6		
Colonization or infection with *Clostridioides difficile *^*c*^	9	5.6	3	3.9		
Clinical characteristics						
Focus of infection					0.014	3 / 1.3
Urogenital	30	18.8	17	22.4		
Skin/ Soft tissue	11	6.9	10	13.2		
Bone	1	0.6	1	1.3		
Abdominal	18	11.3	11	14.5		
Pulmonal	16	10.0	4	5.3		
Central venous catheter	29	18.1	3	3.9		
Others (incl. unclear)	55	34.4	30	39.5		
ICU transfer ^d^	56	35.0	9	11.8	< 0.001	3 / 1.3
Vasopressor ^d^	38	23.8	6	7.9	0.004	3 / 1.3
Mechanical ventilation ^d^	35	21.9	6	7.9	0.009	3 / 1.3
Systolic blood pressure ≤ 100mmHg^d^	82	51.6	31	40.8	0.128	4 / 1.7
Highest body temperature ^e^	38.0	37.2–38.7	38.3	37.1–38.9	0.436	6 / 2.5
Treatment characteristics						
Adequate empirical therapy	125	77.2	61	79.2	0.868	0 / 0.0
Source control achieved ^f^	63	39.4	22	28.9	0.147	3 / 1.3

*Abbreviations*: *ICU* intensive care unit, *IQR* interquartile range, *ESBL* extended-spectrum beta-lactamases, *n* number, *PPI* proton pump inhibitor

^a^in the prior 3 months, ^b^in the prior 12 months, ^c^ in the prior 6 months, ^d^within 24 h of bloodstream infection onset, ^e^within 12 h before or after blood sample, ^f^within 5 days

### Clinical outcomes

In-hospital mortality (15.4% vs 7.8%), new onset renal impairment (13.6% vs 7.8%), and recurrent infection with AmpC-PE (11.7% vs 6.5%) did not differ between the two treatment groups. Fewer neurological adverse events were reported in patients receiving cefepime versus those receiving carbapenems (5.2% vs 21.0%). The overall length of hospital stay was shorter for survivors in the cefepime group (median 17.0 vs 23.0) (Table 2). Uni- and multivariable analyses revealed similar results and were further substantiated by the sensitivity analysis confidence estimates based on robust standard errors (Table 3).

**Table 2:** Outcome characteristics stratified by definite antimicrobial therapy

	Definite carbapenem treatment (*n* = 162)		Definite cefepime treatment (*n* = 77)		*p*-value
	n/ median	%/ IQR	n/ median	%/IQR	
Primary outcomes					
In-hospital death	25	15.4	6	7.8	0.148
New renal impairment	22	13.6	6	7.8	0.281
Neurological adverse events	34	21.0	4	5.2	0.001
Secondary outcomes					
Length of stay (survivors)	23.0	12.0–37.0	17.0	9.0–30.0	0.031
First recurrent infection with AmpC-PE ^a^	19	11.7	5	6.5	0.255
Second recurrent infection with AmpC-PE ^a^	3	15.8^b^	2	40.0^b^	0.270

*Abbreviations*: *AmpC-PE* AmpC-producing Enterobacterales, *IQR* interquartile range, *n* number

^a^in the following 6 months, ^b^out of total first recurrent infection with AmpC-PE

**Table 3:** Associations between definite antimicrobial therapy and the different outcomes (reference definite therapy with carbapenems)

	Univariable			Multivariable^a^			Univariable robust			Multivariable robust^a^		
	OR	95% CI	*p*-value	OR	95% CI	*p*-value	OR	95% CI	*p*-value	OR	95% CI	*p*-value
Primary outcomes												
In-hospital death	.46	.18; 1.18	0.107	.77	.28; 2.18	0.628	.46	.19 1.16	0.101	.77	.27; 2.19	0.630
New renal impairment	.54	.21; 1.39	0.199	.54	.20; 1.47	0.230	.54	.21; 1.41	0.208	.54	.20; 1.48	0.233
Neurological adverse events	.21	.07; .61	0.004	.26	.09; .79	0.018	.21	.07; .61	0.004	.26	.09; .80	0.018
Secondary outcomes												
Length of stay (survivors) ^b^	-27.50	-44.87; -4.61	0.022	-27.48	-45.97; -2.66	0.032	-27.50	-45.59; -3.35	0.028	-27.48	-46.63; -1.46	0.040
First recurrent infection	0.51	0.18; 1.42	0.195	0.69	0.23; 2.05	0.501	0.51	0.18; 1.45	0.206	0.69	0.21; 2.21	0.528
Second recurrent infection ^c^	3.56	0.40; 31.23	0.253	6.64	0.37; 118.18	0.198	3.56	0.38; 33.57	0.268	6.64	0.54; 82.32	0.141

*Abbreviations*: *OR* Odds Ratio, *CI* Confidence interval

^a^complete case analyses (missing co-variables for three episodes)

^b^estimates shown as % change, i.e. (exponentiated coefficients—1)*100

^c^among episodes with a first recurrence

### Microbiological outcomes

Within the following six months, a first recurrent infection with an AmpC-PE occurred after 19 episodes (11.7%) in the carbapenem group compared to five episodes (6.5%) in the cefepime group (*p* = 0.255). A second recurrent AmpC-PE infection occurred after three episodes (1.8%) of the carbapenem group and after two episodes (2.5%) of the cefepime group (*p* = 0.270). The corresponding type of infections are presented in Supplementary table 2. Further microbiological characteristics on the AmpC-PE causing the recurrent infections are included in Supplementary table 3.

During definite antimicrobial treatment of the initial episode of AmpC-PE BSI, seven (4.2%) pathogens in the carbapenem group became intermediate or resistant against any carbapenem compared to two (2.6%) in the cefepime group. Three (1.8%) pathogens in the carbapenem group and one (1.3%) in the cefepime group became intermediate or resistant to cefepime. In the carbapenem group, four (20.0%) pathogens causing a first recurrent infection showed increasing resistance and two (10.0%) decreasing resistance compared to the susceptibility test result of their initial BSI. In the cefepime group as well as for all second recurrent infections], there was no change in the resistance profil between BSI and first recurrent infection (Supplementary table 4).

## Discussion

After excluding ESBL-producing isolates, patients with renal impairment, and patients at risk for neurotoxicity, this cohort study found the use of cefepime versus carbapenems for treating BSI caused by AmpC-PE was not associated with in hospital mortality, recurrent infection, or adverse effects.

These results are consistent with the findings of previous studies reporting cefepime as effective alternative treatment for AmpC-PE infections, and add to the existing evidence supporting cefepime as a safe treatment strategy [4, 6, 21].

Cefepime was not associated with a higher rate of treatment related renal impairment and had significantly less neurological adverse events compared to carbapenems. The latter might be explained by our institutional guidelines cautioning against the use of cefepime in patients at increased risk for side effects of the central nervous system and increased vulnerability to neurological adverse events in case of circulatory instability or need of intensive care. However, our findings are consistent with previous literature indicating that relevant neurological adverse events of cefepime are rare, even in severely ill patients [12]. Furthermore, our study showed that the length of stay in the cefepime group, particularly among survivors, was around 30% shorter compared to the carbapenem group but the respective confidence intervals were wide – ranging from -46% to -3%. Our findings indicate that cefepime may be a valuable alternative to carbapenems, not only as a carbapenem-sparing regimen, but also for potentially improving patient outcomes. These results need to be substantiated in a randomized trial.

Interestingly, our analysis showed that the adequacy of empirical antibiotic treatment did not have a significant effect on outcomes. This finding is consistent with recent literature on the relationship between inadequate empirical therapy and mortality where the authors found no association and referred to residual confounding as a possible explanation [22]. In contrast, inappropriate initial antibiotic treatment is known to be associated with increased hospital mortality in Gram-negative bacteremia complicated by severe sepsis or septic shock [23]. As only 27% of BSI episodes within our cohort required ICU-referral within 12 h of blood sample collection, our finding may be attributable to the majority of patients not presenting with severe sepsis at BSI-onset.

Recurrent infections with an AmpC-PE occurred more frequently among patients treated with carbapenems as compared to patients treated with cefepime; however, this did not reach statistical significance likely due to the low rate of recurrences in this study population. Notably, emergence of resistance of AmpC-PE was more commonly noted after treatment of the initial infection episode with carbapenems. Recently, imipenem has been shown to promote AmpC expression and may induce an adaptive response to carbapenems by regulating key genes involved in the control of efflux pumps and porins, which could lead to a multidrug-resistant profile in clinical isolates, contributing to possible treatment failure [24, 25]. Further studies are needed to compare the potential of different beta-lactams in inducing resistance by induction of AmpC or other resistance mechanisms, such as the up-regulation of efflux pumps or down-regulation of the expression of porins.

The inclusion of a relatively large number of patients with BSI caused by AmpC-PE and the systematic exclusion of ESBL-production among all causative isolates are strengths of this study. In addition, patients treated with cefepime and carbapenems were well balanced regarding baseline characteristics and risk factors.

Our study has some relevant limitations, most importantly its retrospective single center design relying on data collection from electronical medical records. Despite best efforts to minimize potential confounding, residual confounding may have occurred. Furthermore, our findings may only be generalizable to similar healthcare settings and patient populations. Recurrent infections may have been missed in patients subsequently not presenting to our institution. Changes in susceptibility of AmpC-PE recovered in the context of recurrent infections were assessed based on Eucast classifications – changes in MICs were not evaluated as at our institution susceptibility testing is routinely performed using the VITEK® system, which lakes the accuracy to determine precise changes in MIC levels. Duration of inadequate treatment prior to establishment of definitive treatment was not assessed and differences in duration of inadequate treatment may act as an important confounder when assessing differences in outcomes between the two treatment groups. As, however, all positive blood culture results are assessed by the infectious disease consultation service on a daily basis and definite treatment with either cefepime or carbapenems was assigned based on pre-defined criteria as indicated in our institutional guidelines, we consider it unlikely that duration of inadequate treatment would differ between the two groups. We further acknowledge that no sample size calculations were performed a priori and that based on our sample size the study may be underpowered to detect differences between the two treatment groups regarding the outcomes investigated (in line post-hoc power analyses reveals a power of 35.9% of our study to detect a significant difference for mortality between the two treatment groups).

In summary, our study provides further evidence that cefepime is a useful carbapenem-sparing agent for the treatment of AmpC-PE BSI if ESBL-production can be excluded. Importantly, our results indicate that cefepime treatment may not be associated with a negative impact on relevant clinical outcomes, including mortality, recurrent infections, and length of hospital stay. These findings present further evidence to support treating AmpC-PE BSI with cefepime as a safe strategy, particularly in clinically stable patients without initial renal impairment or increased susceptibility to neurological adverse events. Robust randomized controlled trials should be conducted to validate these findings.

### Supplementary Information

Below is the link to the electronic supplementary material.

Supplementary file1 (DOCX 47 KB)

## Data Availability

The datasets generated and analysed during the current study are available from the corresponding author on reasonable request.
