# Supplementary material for: Cefepime versus carbapenems for treatment of AmpC beta-lactamase-producing Enterobacterales bloodstream infections
Source: Eur J Clin Microbiol Infect Dis. 2023 Nov 23;43(2):213–21. doi: 10.1007/s10096-023-04715-5 (PMC10821988; doi:10.1007/s10096-023-04715-5)
Supplement: Supplementary file 1 — Supplementary file1 (DOCX 47 KB) [file 10096_2023_4715_MOESM1_ESM.docx]

**Cefepime versus carbapenems for treatment of AmpC beta-lactamase producing Enterobacterales bloodstream infections**

European Journal of Clinical Microbiology & Infectious Diseases

*Julia Herrmann^1^, Anne-Valérie Burgener-Gasser^1^, Daniel Goldenberger^2^, Jan Roth^1^, Maja Weisser^1^, Pranita D.Tamma^3^, Sarah Tschudin-Sutter^1^*

^1^Division of Infectious Diseases and Hospital Epidemiology, University Hospital Basel and University of Basel, Basel, Switzerland; ^2^Division of Clinical Bacteriology and Mycology, University Hospital Basel and University of Basel, Basel, Switzerland; ^3^Department of Pediatrics, Johns Hopkins University School of Medicine, Baltimore, MD, USA

**Corresponding author**

Prof. Sarah Tschudin-Sutter, MD, MSc

Division of Infectious Diseases and Hospital Epidemiology

University Hospital Basel, Petersgraben 4, CH-4031 Basel, Switzerland

Phone: +41 61 265 25 25

E-mail: [sarah.tschudin@usb.ch](mailto:sarah.tschudin@usb.ch)

# Supplementary Material

**Microbiological data**

Species of AmpC producing Enterobacterales included *Citrobacter braakii (C. freundii group), Citrobacter freundii, Citrobacter freundii group, Citrobacter sedlakii (C. freundii group), Citrobacter youngae (C. freundii group), Enterobacter asburiae (E. cloacae group), Enterobacter cancerogenus, Enterobacter cloacae, Enterobacter cowanii, Enterobacter species, Enterobacter cloacae group, Enterobacter intermedius, Hafnia alvei, Klebsiella aerogenes (*previously *Enterobacter), Morganella morganii, Providencia alcalifaciens, Providencia rettgeri, Providencia stuartii, Providencia species, Serratia ficaria, Serratia fonticola, Serratia liquefaciens, Serratia marcescens, Serratia odorifera, Serratia plymuthica, Serratia liquefaciens group, Serratia species* and *Serratia rubidaea*

Supplementary table 1: Microbiological characteristics of AmpC-PE bloodstream infections stratified by definite antimicrobial therapy and AmpC-PE (n= 283)

|  | Definite carbapenem  treatment  (n = 171) | | Definite cefepime  treatment  (n = 80) | | Definite pip./taz.  treatment  (n = 15) | | Definite  other  treatment  (n = 17) | | Total  (n = 283) | |
| --- | --- | --- | --- | --- | --- | --- | --- | --- | --- | --- |
|  | n | % | n | % | n | % | n | % | n | % |
| AmpC-PE Species |  |  |  |  |  |  |  |  |  |  |
| *Citrobacter freundii* | 3 | 1.8 | 1 | 1.3 | 0 | 0.0 | 1 | 5.9 | 5 | 1.9 |
| *Citrobacter freundii* group | 18 | 10.5 | 2 | 2.6 | 1 | 6.7 | 2 | 11.8 | 23 | 8.5 |
| Other *Citrobacter* spp.^1^ | 0 | 0.0 | 0 | 0.0 | 0 | 0.0 | 0 | 0.0 | 0 | 0.0 |
| *Enterobacter asburiae* (*E. cloacae* group) | 0 | 0.0 | 1 | 1.3 | 0 | 0.0 | 0 | 0.0 | 1 | 0.4 |
| *Enterobacter cloacae* | 2 | 1.2 | 1 | 1.3 | 1 | 6.7 | 0 | 0.0 | 3 | 1.1 |
| *Enterobacter cloa*cae group | 76 | 44.4 | 31 | 40.3 | 0 | 0.0 | 6 | 35.3 | 114 | 42.2 |
| Other *Enterobacter* spp.^2^ | 0 | 0.0 | 0 | 0.0 | 0 | 0.0 | 0 | 0.0 | 0 | 0.0 |
| *Hafnia alvei* | 2 | 1.2 | 0 | 0.0 | 0 | 0.0 | 0 | 0.0 | 2 | 0.7 |
| *Klebsiella aerogenes* (previously Enterobacter) | 15 | 8.8 | 11 | 14.3 | 7 | 46.7 | 6 | 35.3 | 39 | 14.4 |
| *Morganella morganii* | 10 | 5.8 | 6 | 7.8 | 2 | 13.3 | 1 | 5.9 | 19 | 7.0 |
| *Providencia rettgeri* | 1 | 0.6 | 1 | 1.3 | 0 | 0.0 | 0 | 0.0 | 2 | 0.7 |
| Other *Providencia* spp.^3^ | 0 | 0.0 | 0 | 0.0 | 0 | 0.0 | 0 | 0.0 | 0 | 0.0 |
| *Serratia marcescens* | 44 | 25.7 | 25 | 32.5 | 4 | 26.7 | 1 | 5.9 | 74 | 27.4 |
| *Serratia liquefaciens* group | 0 | 0.0 | 1 | 1.3 | 0 | 0.0 | 0 | 0.0 | 1 | 0.4 |
| Other *Serratia* spp.^4^ | 0 | 0.0 | 0 | 0.0 | 0 | 0.0 | 0 | 0.0 | 0 | 0.0 |
| ESBL producing bacteria | 6 | 3.5 | 0 | 0.0 | 0 | 0.0 | 0 | 0.0 | 6 | 2.2 |
| *Citrobacter freundii* | 1 | 0.6 | 0 | 0.0 | 0 | 0.0 | 0 | 0.0 | 1 | 0.4 |
| Other *Citrobacter* spp.^5^ | 0 | 0.0 | 0 | 0.0 | 0 | 0.0 | 0 | 0.0 | 0 | 0.0 |
| *Enterobacter cloacae* | 0 | 0.0 | 0 | 0.0 | 0 | 0.0 | 0 | 0.0 | 0 | 0.0 |
| *Enterobacter cloacae* group | 4 | 2.3 | 0 | 0.0 | 0 | 0.0 | 0 | 0.0 | 4 | 1.5 |
| Other *Enterobacter* spp.^6^ | 0 | 0.0 | 0 | 0.0 | 0 | 0.0 | 0 | 0.0 | 0 | 0.0 |
| *Hafnia alvei* | 0 | 0.0 | 0 | 0.0 | 0 | 0.0 | 0 | 0.0 | 0 | 0.0 |
| *Klebsiella aerogenes* (previously Enterobacter) | 0 | 0.0 | 0 | 0.0 | 0 | 0.0 | 0 | 0.0 | 0 | 0.0 |
| *Morganella morganii* | 0 | 0.0 | 0 | 0.0 | 0 | 0.0 | 0 | 0.0 | 0 | 0.0 |
| *Providencia* spp.^7^ | 0 | 0.0 | 0 | 0.0 | 0 | 0.0 | 0 | 0.0 | 0 | 0.0 |
| *Serratia marcescens* | 1 | 0.6 | 0 | 0.0 | 0 | 0.0 | 0 | 0.0 | 1 | 0.4 |
| Other *Serratia* spp.^8^ | 0 | 0.0 | 0 | 0.0 | 0 | 0.0 | 0 | 0.0 | 0 | 0.0 |
| Carbapenemase producing bacteria | 0 | 0.0 | 0 | 0.0 | 0 | 0.0 | 0 | 0.0 | 0 | 0.0 |
| Initial resistance (I and R combined) |  |  |  |  |  |  |  |  |  |  |
| None | 111 | 64.9 | 53 | 66.3 | 12 | 80.0 | 15 | 88.2 | 191 | 67.5 |
| Ceftriaxon | 45 | 26.3 | 14 | 17.5 | 1 | 6.7 | 1 | 5.8 | 61 | 21.5 |
| Ceftazidim | 43 | 25.1 | 14 | 17.5 | 1 | 6.7 | 1 | 5.8 | 59 | 20.8 |
| Cefepime | 19 | 11.1 | 0 | 0.0 | 0 | 0.0 | 0 | 0.0 | 20 | 7.0 |
| Piperacillin/tazobactam | 32 | 20.8 | 14 | 17.6 | 0 | 0.0 | 0 | 0.0 | 48 | 16.9 |
| Ertapenem | 11 | 6.4 | 3 | 3.8 | 0 | 0.0 | 0 | 0.0 | 15 | 5.3 |
| Imipenem | 7 | 4.1 | 7 | 8.8 | 2 | 13.3 | 1 | 5.9 | 17 | 6.1 |
| Meropenem | 2 | 1.2 | 0 | 0.0 | 0 | 0.0 | 1 | 5.9 | 3 | 1.1 |
| *C. braakii, C. sedlakii, C. youngae*  2 *E. cancerogenus, E. cowanii, E. species, E. intermedius*  3 *P. alcalifaciens, P. stuartii, P. species*  4 *S. ficaria, S. fonticola, S. liquefaciens, S. odorifera, S. plymuthica, S. species, S. rubidaea*  5 *C. braakii, C. sedlakii, C. youngae, C. freundii group*  6 *E. asburiae, E. cancerogenus, E. cowanii, E. species, E. intermedius,*  7 *P. alcalifaciens, P. stuartii, P. species, P. rettgeri*  8 *S. ficaria, S. fonticola, S. liquefaciens, S. liquefaciens group, S. odorifera, S. plymuthica, S. species, S. rubidaea*  *Abbreviations: AmpC-PE, AmpC-producing Enterobacterales; ESBL, extended-spectrum beta-lactamases; I, intermediate; Pip./Taz, piperacillin/tazobactam; R, resistant* | | | | | | | | | | |

Supplementary table 2: Occurrence of recurrent infections stratified by definite antimicrobial therapy and episode (n= 270)

|  | Definite carbapenem treatment (n = 162) | | Definite cefepime treatment  (n = 77) | |
| --- | --- | --- | --- | --- |
|  | n | % | n | % |
| First recurrent infection with AmpC-PE^1^ | **19** | **11.7** | **5** | **6.5** |
| Type of first recurrent infection^1^ |  |  |  |  |
| Blood sample infection | 7 | 4.3 | 2 | 2.6 |
| Pneumonia | 6 | 3.7 | 1 | 1.3 |
| Osteomyelitis | 0 | 0.0 | 1 | 1.3 |
| Urinary tract infection | 2 | 1.2 | 1 | 1.3 |
| Soft tissue infection | 1 | 0.6 | 0 | 0.0 |
| Surgical site infection | 3 | 1.9 | 0 | 0.0 |
| Intraabdominal infection | 0 | 0.0 | 0 | 0.0 |
| Others | 0 | 0.0 | 0 | 0.0 |
| Second recurrent infection with AmpC-PE^1^ | **3** | **15.8** | **2** | **40.0** |
| Type of second recurrent infection^1^ |  |  |  |  |
| Blood sample infection | 1 | 33.3 | 1 | 50.0 |
| Pneumonia | 0 | 0.0 | 0 | 0.0 |
| Osteomyelitis | 0 | 0.0 | 0 | 0.0 |
| Urinary tract infection | 0 | 0.0 | 1 | 50.0 |
| Soft tissue infection | 0 | 0.0 | 0 | 0.0 |
| Surgical site infection | 1 | 33.3 | 0 | 0.0 |
| Intraabdominal infection | 0 | 0.0 | 0 | 0.0 |
| Others | 1 | 33.3 | 0 | 0.0 |
| *^1^ in the following 6 months*  *Abbreviations: AmpC-PE, AmpC-producing Enterobacterales* | | | | |

Supplementary table 3: Microbiological characteristics of AmpC-PE causing first recurrent infections stratified by definite antimicrobial therapy and AmpC-PE (n =283)

|  | Definite carbapenem treatment  (n = 171) | | Definite cefepime treatment  (n = 80) | |
| --- | --- | --- | --- | --- |
|  | n | % | n | % |
| AmpC-PE species causing first recurrent infection^1^ | 20 | 11.7 | 5 | 6.3 |
| *Citrobacter freundii* | 1 | 0.6 | 0 | 0.0 |
| *Citrobacter freundii* group | 3 | 1.8 | 0 | 0.0 |
| Other *Citrobacter* spp.^2^ | 0 | 0.0 | 0 | 0.0 |
| *Enterobacter cloacae* | 0 | 0.0 | 1 | 1.3 |
| *Enterobacter cloacae* group | 8 | 4.7 | 2 | 2.5 |
| Other *Enterobacter* spp.^3^ | 0 | 0.0 | 0 | 0.0 |
| *Hafnia alvei* | 0 | 0.0 | 0 | 0.0 |
| *Klebsiella aerogenes* (previously *Enterobacter*) | 2 | 1.2 | 1 | 1.3 |
| Morganella morganii | 0 | 0.0 | 0 | 0.0 |
| *Providencia* spp.^4^ | 0 | 0.0 | 0 | 0.0 |
| *Serratia liquefaciens* | 1 | 0.6 | 0 | 0.0 |
| *Serratia marcescens* | 5 | 2.9 | 1 | 1.3 |
| Other *Serratia* spp.^5^ | 0 | 0.0 | 0 | 0.0 |
| ESBL-producing bacteria causing first recurrent infection^1^ | 4 | 2.3 | 0 | 0.0 |
| *Citrobacter* spp.^6^ | 0 | 0.0 | 0 | 0.0 |
| *Enterobacter cloacae* | 0 | 0.0 | 0 | 0.0 |
| *Enterobacter cloacae* group | 4 | 2.3 | 0 | 0.0 |
| Other *Enterobacter* spp.^7^ | 0 | 0.0 | 0 | 0.0 |
| *Hafnia alvei* | 0 | 0.0 | 0 | 0.0 |
| *Klebsiella aerogenes* (previously *Enterobacter*) | 0 | 0.0 | 0 | 0.0 |
| *Morganella morganii* | 0 | 0.0 | 0 | 0.0 |
| *Providencia* spp.^8^ | 0 | 0.0 | 0 | 0.0 |
| *Serratia* spp.^9^ | 0 | 0.0 | 0 | 0.0 |
| Carbapenemase-producing bacteria causing first recurrent infection^1^ | 0 | 0.0 | 0 | 0.0 |
| Initial resistance of first recurrent infection^1^ (I and R combined) | | | | |
| None | 10 | 5.8 | 3 | 3.8 |
| Cefepime | 6 | 3.5 | 0 | 0.0 |
| Piperacillin/Tazobactam | 6 | 3.5 | 2 | 2.5 |
| Ertapenem | 2 | 1.2 | 0 | 0.0 |
| Imipenem | 1 | 0.6 | 0 | 0.0 |
| Meropenem | 1 | 0.6 | 0 | 0.0 |
| *In the following 6 months*  *2 C. braakii, C. sedlakii, C. youngae*  *3 E. asburiae, E. cancerogenus, E. cowanii, E. species, E. intermedius,*  *4 P. alcalifaciens, P. stuartii, P. species, P. rettgeri54 S. ficaria, S. fonticola,, S. liquefaciens group, S. odorifera, S. plymuthica, S. species, S. rubidaea*  *5 C. braakii, C. freundii, C. freundii group, C. sedlakii, C. youngae*  *^6^ C. braakii, C. freundii, C. freundii group, C. sedlakii, C. youngae*  *^7^ E. asburiae, E. cancerogenus, E. cowanii, E. species, E. intermedius,*  *^8^ P. alcalifaciens, P. stuartii, P. species, P. rettgeri*  *^9^ S. ficaria, S. fonticola, S. liquefaciens,, S. liquefaciens group, S. marcescens, S. odorifera, S. plymuthica, S. species, S. rubidaea*  *Abbreviations: AmpC-PE, AmpC-producing Enterobacterales; BSI, bloodstream infection; I or R, intermediate or resistant* | | | | |

Supplementary table 4: Microbiological outcome of resistance (I or R) stratified by definite antimicrobial therapy and AmpC-PE (n = 283)

|  | Definite carbapenem treatment  (n = 171) | | Definite cefepime treatment  (n = 80) | |
| --- | --- | --- | --- | --- |
|  | n | % | n | % |
| Developed resistance under definite treatment of BSI (I and R combined) | | | | |
| None | 153 | 89.5 | 72 | 90.00 |
| Cefepime | 3 | 1.8 | 1 | 1.3 |
| Piperacillin/Tazobactam | 2 | 1.2 | 1 | 1.3 |
| Ertapenem | 3 | 1.8 | 2 | 2.6 |
| Imipenem | 3 | 1.8 | 0 | 0.0 |
| Meropenem | 1 | 0.6 | 0 | 0.0 |
| Unknown | 4 | 2.3 | 3 | 3.8 |
|  | **Definite carbapenem treatment**  **(n = 20)** | | **Definite cefepime treatment**  **(n = 5)** | |
|  | n | % | n | % |
| Change in resistance between BSI and first recurrent infection^1^ | | | | |
| None | 9 | 45.0 | 4 | 80.0 |
| Decrease | 2 | 10.0 | 0 | 0.0 |
| Cefepime I or R | 1 | 5.0 | 0 | 0.0 |
| Pip/Taz I or R | 1 | 5.0 | 0 | 0.0 |
| Increase | 4 | 20.0 | 0 | 0.0 |
| Cefepime I or R | 2 | 10.0 | 0 | 0.0 |
| Pip/Taz I or R | 2 | 10.0 | 0 | 0.0 |
| ESBL | 1 | 5.0 | 0 | 0.0 |
| Different pathogen | 5 | 25.0 | 1 | 20.0 |
| Developed resistance under treatment of first recurrent infection^1^ | 0 | 0.0 | 0 | 0.0 |
|  | **Definite carbapenem treatment**  **(n = 3)** | | **Definite cefepime treatment**  **(n = 2)** | |
|  | n | % | n | % |
| Change in resistance between BSI and second recurrent infection^1^ | | | | |
| None | 1 | 33.3 | 2 | 100.0 |
| Decrease | 0 | 0.0 | 0 | 0.0 |
| Increase | 0 | 0.0 | 0 | 0.0 |
| Different pathogen | 2 | 66.6 | 0 | 0.0 |
| *In the following 6 months*  *Abbreviations: AmpC-PE, AmpC-producing Enterobacterales; BSI, bloodstream infection; I or R, intermediate or resistant* | | | | |
